# Blood tests in primary care: A qualitative study of communication and decision‐making between doctors and patients

**DOI:** 10.1111/hex.13564

**Published:** 2022-07-19

**Authors:** Jessica Watson, Penny F. Whiting, Chris Salisbury, William T. Hamilton, Jonathan Banks

**Affiliations:** ^1^ Centre for Academic Primary Care, Population Health Sciences University of Bristol Bristol UK; ^2^ University of Exeter Medical School Exeter UK; ^3^ National Institute for Health Research Applied Research Collaboration West (NIHR ARC West) Bristol UK

**Keywords:** blood tests, doctor–patient relationship, paternalism, patient engagement, primary health care, qualitative research, shared decision‐making

## Abstract

**Objective:**

Blood tests are commonly used in primary care as a tool to aid diagnosis, and to offer reassurance and validation for patients. If doctors and patients do not have a shared understanding of the reasons for testing and the meaning of results, these aims may not be fulfilled. Shared decision‐making is widely advocated; yet, most research focusses on treatment decisions rather than diagnostic decisions. The aim of this study was to explore communication and decision‐making around diagnostic blood tests in primary care.

**Methods:**

Qualitative interviews were undertaken with patients and clinicians in UK primary care. Patients were interviewed at the time of blood testing, with a follow‐up interview after they received test results. Interviews with clinicians who requested the tests provided paired data to compare clinicians' and patients' expectations, experiences and understandings of tests. Interviews were analysed thematically using inductive and deductive coding.

**Results:**

A total of 80 interviews with 28 patients and 19 doctors were completed. We identified a mismatch in expectations and understanding of tests, which led to downstream consequences including frustration, anxiety and uncertainty for patients. There was no evidence of shared decision‐making in consultations preceding the decision to test. Doctors adopted a paternalistic approach, believing that they were protecting patients from anxiety.

**Conclusion:**

Patients were not able to develop informed preferences and did not perceive that choice is possible in decisions about testing, because they did not have sufficient information and a shared understanding of tests. A lack of shared understanding at the point of decision‐making led to downstream consequences when test results did not fulfil patients' expectations. Although shared decision‐making is recommended as best practice, it does not reflect the reality of doctors' and patients' accounts of testing; a broader model of shared understanding seems to be more relevant to the complexity of primary care diagnosis.

**Patient or Public Contribution:**

A patient and public involvement group comprising five participants with lived experience of blood testing in primary care met regularly during the study. They contributed to the development of the research objectives, planning recruitment methods, reviewing patient information leaflets and topic guides and also contributed to discussion of emerging themes at an early stage in the analysis process.

## 1 INTRODUCTION

Blood tests are commonly used in primary care, fulfilling important diagnostic, prognostic and therapeutic purposes.^1^ Qualitative studies with doctors and patients have shown that blood tests also fulfil psychosocial roles in the consultation, for example, providing reassurance, helping to build an empathic connection and providing validation for patients' symptoms.^2^, ^3^, ^4^ Inflammatory markers are a type of blood test commonly used for the diagnosis and monitoring of infections, autoimmune conditions and cancers,^5^ and as a nonspecific screening test for patients with unexplained symptoms.^6^ Inflammatory markers rarely offer a specific diagnosis, but can offer ‘clues’. False‐positive inflammatory markers can lead to increased rates of follow‐on consultations, blood tests and referrals.^7^ It is therefore important that doctors and patients have a shared understanding of the reasons for inflammatory marker testing and the potential pitfalls of testing.

Shared decision‐making is a process in which patients and clinicians work together to make decisions based on evidence and patients' preferences, and is widely recommended as ‘best practice’.^8^ Awareness of the importance of shared decision‐making is widespread; yet, most research focusses on treatment decisions rather than diagnostic testing. Where research into diagnostic testing does exist, it mostly focusses on specific tests, for example, prostate‐specific antigen for prostate cancer.^9^

The aim of this study was to examine communication and decision‐making around inflammatory marker blood tests in primary care. Although we examined testing decisions through a lens of shared decision‐making, we chose not to limit our analysis to shared decision‐making, as we wished to consider the process of communication from the initial consultation to test results.

## 2 METHODS

This study used qualitative interviews with doctors and patients. A participating patient's blood test represented a ‘case’, which was examined by interviewing (a) the patient at the time of testing; (b) the patient after the test results had been obtained; and (c) the doctor who requested the test.

### 2.1 Recruitment

UK general practices were recruited with the support of the West of England Clinical Research Network. Six practices were recruited to include urban and rural practices, and a range of population characteristics, including deprivation, age and ethnicity. All general practitioners (GPs) in participating practices were invited to participate, including locums, salaried GPs and partners.

Patients were eligible to participate if they were aged >18 years, having inflammatory marker blood tests requested by participating GPs and able to speak English sufficiently for interviews. Patients were sampled by gender, age and socioeconomic status.

Eligible patients were offered study information at the time of testing by their GP or phlebotomist. Interviews were conducted face to face at participants' GP practice at the time of blood testing or soon afterwards at the University of Bristol according to the patient's convenience if preferred. A follow‐on telephone interview with the patient was arranged 1–2 weeks later, to explore the communication of the test results.

Participants were informed that the interviewer was a GP; it was emphasized that the interviews were nonjudgemental, and were focussed on exploring communication around testing, not on scrutinizing the clinical decision‐making.

After patient recruitment, the GP who had requested the blood tests was contacted to arrange a telephone interview. Each GP could complete a maximum of two interviews (about different patients), to maximize the range of GPs.

### 2.2 Interviews

Interviews were carried out by J. W., a practising GP with experience and training in qualitative research methodology. Interviews were semi‐structured, using topic guides based on the research questions, but flexible enough to allow exploration of issues raised by the participant. The topic guide (see the Supporting Information Material) was adapted iteratively during the study, using information emerging in early interviews to inform subsequent interviews. The initial patient interviews focussed on the patients' understanding of the rationale for testing, their expectations of testing and the communication around the decision to test. Follow‐up patient interviews focussed on patients' experiences of receiving and interpreting their test results. The GP interviews allowed comparison of patient and GP perspectives of reasons for testing, expectations of tests and communication around testing. GPs undertook the interviews with access to the patients' electronic medical records at the time of interviewing as an aide memoire.

Interviews were continued until a diverse sample had been recruited and data saturation was achieved, meaning that the topic guide was stable, with no new codes arising.^10^

### 2.3 Analysis

Audio recordings were transcribed verbatim by an experienced transcriber. Analysis began when the first transcripts were available, so that data collection and analysis were conducted concurrently. Transcripts were analysed using thematic analysis, involving a mixture of inductive and deductive coding and constant comparison.^11^

Two members of the research team (J. W. and J. B.) independently reviewed four transcripts to develop an initial coding framework that reflected the research objectives. This framework was adapted following discussions with the study team and tested on a further three transcripts by J. W. and J. B. J. W. then took responsibility for ongoing coding and categorization of the data, using NVivo qualitative data management software. Categories of data and thematic relationships were identified and written up as descriptive and interpretive accounts.

Ethics approvals were obtained from the proportionate review subcommittee of the London—Hamstead NHS Research Ethics committee (REC 19/LO/0405).

## 3 RESULTS

The characteristics of the 28 patients and 19 GPs recruited from 6 practices are summarized in Tables 1 and 2. Patients reflected a range of deprivation, age and ethnicity. The proportion of female patients recruited (64%) is in keeping with the gender balance of patients receiving inflammatory marker blood tests.^7^ Participating clinicians were 68% GP partners, 26% salaried GP and 74% female, with a range of years of experience. Eighty interviews were carried out: 26 GP interviews and 54 patient interviews; most patients were interviewed twice, and some GPs were interviewed about two different patients.

**Table 1:** Characteristics of the participating patients (*n* = 28)

Characteristic	*n* (%)
Gender	
Female	18 (64%)
Male	10 (36%)
Ethnicity	
White British	23 (82%)
BAME	3 (11%)
Other non‐British	2 (7%)
Age group	
18–24	8 (29%)
25–34	3 (11%)
35–44	3 (11%)
45–54	3 (11%)
55–64	3 (11%)
65–74	1 (4%)
75+	7 (25%)
Socioeconomic status (based on postcode IMD)	
1 (Most deprived)	2 (7%)
2	5 (18%)
3	2 (7%)
4	4 (14%)
5	0 (0%)
6	2 (7%)
7	2 (7%)
8	3 (11%)
9	2 (7%)
10 (Most affluent)	1 (4%)
Postcode unavailable	5 (18%)

Abbreviation: IMD, index of multiple deprivation.

**Table 2:** Characteristics of the participating GPs (*n* = 19)

Characteristic	*n* (%)
Gender	
Female	14 (74%)
Male	5 (26%)
Type of GP	
Partner	13 (68%)
Salaried	5 (26%)
Locum	1 (5%)
Years of experience	
0–5 years	5 (26%)
5–10 years	2 (11%)
10–20 years	8 (42%)
20+ years	4 (21%)

Abbreviation: GP, general practitioner.

Four main themes were identified: expectations of testing; patient involvement in decision‐making; information sharing; and blood testing and paternalism in the doctor–patient relationship. Within each theme, doctors' and patients' perceptions are compared, using paired quotes where possible, to demonstrate differences in expectations and understanding within a single clinical encounter.

### 3.1 Expectations of testing

Following the decision to test, patients reflected on their expectations. Few patients directly requested tests, but most saw them as ‘a good thing’. Patients saw tests as a way of moving forward with their problem, and saw testing as a sign that the doctor was taking their symptoms and concerns seriously. Patients had high expectations of their tests; they hoped that tests would provide answers and solutions for their symptoms. For some patients, particularly those with unexplained symptoms, testing offered the promise of meaning and validation for their symptoms.

I think it will make me feel a bit better knowing that it's not just me like taking care of myself badly and there's actually a reason why I might feel the way I do sometimes. (Patient 17, female, 18–24 years, tiredness symptoms)

In contrast, doctors tended to have lower expectations of testing, with a panel or battery of routine tests seen as a useful tool to help rule out serious causes of symptoms, or provide clues rather than clear‐cut diagnoses.

Probably most of ours have got irritable bowel but we sort of need to make sure we've excluded those other things first. (Doctor 15, female, GP partner, 10–20 years' experience)

Doctors' expectations were shaped by their awareness of the limitations and potential pitfalls of testing, although this was rarely shared with patients.

The differences between patients' and doctors' expectations are illustrated when looking at the paired data. For example, Patient 18, who had unexplained joint and muscle pains, expected the blood tests to give answers as to the cause of her symptoms, whereas her doctor expected the results to be normal.

**Table:** 

*I think I was pretty much expecting them to be normal ‘cos I didn't treat her or anything at the time’*. (Doctor 18, male, GP partner, 0–5 years' experience)	*Well just that he would discover what it was and confirm what it was and give me some help*. (Patient 18, female, ≥75 years)

Some doctors were aware of this mismatch in expectations, and therefore tried to discuss and share their expectations with patients to pre‐empt and prepare patients for the possibility of a normal test result.

What I normally do say if I think they're going to be normal, I normally try and pre‐empt that by saying to patients I expect they'll be normal but that will be great and reassuring. (Doctor 23, female, salaried GP, 10–20 years' experience)

### 3.2 Patient involvement in decision‐making

Most decisions to order a blood test were led by doctors, with no examples of the doctor involving the patient in a shared decision to test in any of the interviews. Although some of the patients thought that they had shared the decision, their description of events showed that the doctor had made the decision and the patient agreed or acquiesced without evidence of the patient being involved in decision‐making. No patients recalled being offered alternative tests or the option of no testing, which is generally accepted to be a prerequisite for shared decision‐making. Patients did not perceive blood tests to be a decision where options or choices were possible and, as a result, there was a lack of demand from patients for shared decision‐making.

I think you can always say, ‘no, I don't want it done’, but then if you've got that condition, I mean what choice do you have really? (Patient 4, female, 79 years, headache symptoms)

In three atypical cases, patients had asked their doctor directly for blood tests; all had previous abnormal blood test results that they wanted to recheck or monitor. One of the patients expressed frustration that they needed the doctor's ‘permission’, reflecting the imbalance in power and control over decisions around blood tests.

I mean I don't see any reason why I can't phone the receptionist and say I want a blood test and she'll say well I can't book you in because you haven't had permission from the doctor. (Patient 24, male, 79 years, tiredness symptom, previous anaemia)

### 3.3 Information sharing

Overall, there was a lack of awareness amongst patients about which tests they were having done and why, with less than half of the patients interviewed perceiving that they knew the reason for testing. Although patients mostly acquiesced to doctors' decision‐making, the lack of information sharing around blood tests was perceived as less acceptable by patients.

You walk out and you see somebody, oh what have, you know, you'll see a friend and they'll say what have you been in there for? Had to have blood tests. What for? Don't really know. And you don't… I sometimes think, like I said, it's like the Secret Service, because the doctors tend to, I don't know. How can I put it? It's almost like they kind of shut you out, you‐ Oh we'll just check your levels. (Patient 9, female, 65–74 years, joint symptoms)

After testing, most patients were told whether their tests were normal or abnormal, but few knew the actual results. One of the patients interviewed highlighted the fact that they had not received their results, but only the doctors' interpretation of their results, challenging the assumption that patients should rely on doctors' authority and interpretation.

I mean you say you've got your results back; I haven't got my results back, I've got a doctor's interpretation of my results. (Patient 23, female, 18–24 years, pelvic symptoms)

Not only did patients receive limited information about their results, some patients who had borderline results (outside of the normal range, but not deemed clinically relevant) were unaware of these findings and were under the impression that their tests were completely normal.

**Table:** 

*Her plasma viscosity was 1.87 so a little bit raised, there or thereabouts*. (Doctor for Patient 2, male, GP partner, 20+ years' experience)	*Well, he just said the blood tests came back and it was perfectly normal. No problem, he said no worries at all*. (Patient 2, female, ≥75 years, joint symptoms)

Some doctors reflected openly on this, with a perception that withholding or not openly sharing information about these ‘minor variations’ was justifiable to protect patients from anxiety, reflecting a paternalistic approach.

The difficulty with that is that there are lots of minor variations… you see all the red exclamation marks that come up when you get your blood results back and whilst we know that the vast majority of those are nothing to be concerned about and we would file that as a normal result or satisfactory, it could cause a lot of concern for patients potentially. (Doctor 8, male, GP partner, 0–5 years' experience)

Whilst the majority of patients with normal results received limited information about the meaning of their test results, all patients who had abnormal results received some explanation, and were often reassured by these abnormal results, even when the abnormalities picked up were unlikely to be related to the presenting problem.

It was like oh I have a surprise deficiency. (laughs) Obviously I'm really pleased that that was found. (Patient 23, female, 18–24 years, pelvic symptoms, incidental vitamin D deficiency)

Patients' response to their test results was linked to their expectations of testing. The fact that the doctor had recommended testing in the first place could generate an expectation of finding an underlying cause for the symptoms. For some patients, this meant that instead of being reassured by normal test results, this generated a perception that further tests were needed. Most symptomatic patients with normal results felt some degree of disappointment that the cause for their symptoms had not been found. Some also felt that normal test results seemed to invalidate their experiences, making them feel as if they were being dismissed or written off.

It's semi‐frustrating because you think well that's another thing that doesn't give me the answer then, that's another reason for them to go there's nothing wrong with you. I definitely don't feel that a normal result is a win for me by any means and I can't continue to feel the way that I do so I'm just going to have to keep on looking into it. (Patient 21, female, 25–34 years, abdominal symptoms)

In contrast, most doctors perceived that normal results were reassuring to patients and often assumed that less communication and explanation of normal results was needed compared to abnormal results.

I've kind of left the ball in her court now whereas I think if they were raised and shown signs of inflammation then I would probably make an active plan for follow‐up. (Doctor 18, male, GP partner, 0–5 years' experience)

The problem with this mismatch between doctors' and patients' perceptions of normal results is illustrated in the paired data, as shown by Case 4, a patient with headaches and joint symptoms. The doctor assumes that the test results have provided reassurance for the patient, whereas the patient is worried that ‘nobody knows why’ they still have symptoms.

**Table:** 

*So yeah, I think they [test results] were reassuring for the patient and they were reassuring for me and the rheumatologist that we weren't missing anything potentially serious*. (Doctor for Patient 4, male, GP partner, 10–20 years of experience)	*Well I'm, you know, I get worried really as to why I'm getting these things and nobody knows why. Obviously the blood tests can't be showing anything. I mean I've had MRI scan a while back, but nothing came of that really. I'm just a bit worried as to why I'm getting these things*. (Patient 4, female, ≥75 years, headache symptoms)

There were some positive examples of doctors who were able to build a shared understanding and expectations of tests through open communication and explanation of normal results. For example, Case 6 was a patient with joint symptoms:

So I said to her if they're completely normal I'll be very happy that it's not this condition. If they are very raised, I will phone you and we'll treat you with steroids but I expect they may well be normal. So I kind of gave her the expectation that they probably would be normal and I phoned her and what I've written is ‘phoned to reassure inflammatory markers normal, she's happy to wait and see how things go’. (Doctor 6, female, GP partner, 5–10 years' experience)

The patient, in this case, reflected on the importance of shared understanding and its place in building an open and clear framework of communication between doctor and patient.

I think as long as people know what the tests are for, it's not just oh we'll do a blood test. It's just making people aware of what they're testing for and then people can understand… because blood tests don't really mean anything unless you know what it's for does it really? (Patient 6, female, 45–54 years, joint symptoms)

### 3.4 Blood testing and paternalism in the doctor–patient relationship

Overall, there was a lack of open communication around blood tests. Some doctors perceived that testing was an area where a more paternalistic approach was justified:

I don't find patients ask terribly much about what the blood tests are that we're testing for… I don't have a massive feeling that they want to know terribly much more but maybe I'm wrong about that, I don't know… I think that's still one of those areas of being a GP that patients just trust you're going to ask for the right tests. (Doctor 15, female, GP partner, 10–20 years' experience)

The perception that testing is ‘still one of those areas’ seems to reflect an awareness amongst GPs of the changes over time in medical decision‐making, and the gradual move away from paternalism. Some doctors reflected on this during the interviews and felt that this was an area where they could try to improve their practice.

I'm probably not as good as I might be at sharing that decision, so it's probably more mine actually but I think I will reflect on that. Yes, I'm probably, you know, I think our GP trainees and our trainers are very good at doing shared decision‐making. I'm probably perhaps a bit more old‐fashioned in my approach. (Doctor 6, female, GP partner, 5–10 years' experience)

There was a perception among both doctors and patients that sharing information about testing was the right thing to do ‘in an ideal world’, but multiple barriers to information sharing were identified. Both doctors and patients felt that blood tests were complex and too technical for patients to fully understand. There was a lack of shared language and a lack of resources for sharing information about tests, which meant that even when doctors tried to share information, patients did not always understand or retain information about their blood tests. In the overall context of the consultation, tests were perceived by GPs as relatively trivial interventions and were therefore low priority for information sharing; time and workload pressures added further barriers to a shared understanding of tests.

Doctors considered that protecting patients from undue anxiety was sometimes a justification for withholding information about blood tests. Rather than giving details of which tests were being done, or what was being tested for, they therefore used more general terms, or alluded to possible serious diagnoses.

I probably sort of don't say exactly what I'm looking for, maybe saying make sure there's nothing else worrying going on is probably what I'd be saying more. I think a lot of people tend to understand what that means. I mean a lot of people are worried about cancers and things like that. (Doctor 24, female, salaried GP, 0–5 years' experience)

However, even when these possible serious diagnoses were unspoken, or only alluded to in the consultation, patients used guesswork or tacit knowledge to infer the possibilities, which in itself could provoke anxiety.

Interviewer: What do you think they might be looking for in those tests?

Patient: Something in the blood, like this time I thought maybe because I've got lumps in my neck, I thought maybe leukaemia ‘cos I know that's something, but again that's just a guess’.

Interviewer: Was there anything like that mentioned?

Patient: No. It was just to look out for symptoms like night sweats, weight loss and just feeling unwell. (Patient 12, female, 25–34 years, neck lump symptom)

Although information about testing was sometimes withheld by doctors to prevent patient anxiety, this was not reflected in the patient interviews; in fact, patients generally perceived that a lack of information sharing was more likely to provoke anxiety.

I worry more by not knowing. I do personally. I prefer to know. I think right, I'm sort of like—then I'm prepared, aren't I? I can sort of get all my ducks in a row. (Patient 9, female, 65–74 years, joint symptoms)

## 4 DISCUSSION

These results demonstrate a mismatch between patients' and doctors' expectations and understanding of blood tests. None of those interviewed described a shared decision‐making process around testing, nor did patients perceive blood tests to be a decision where options or choices were possible. Furthermore, some patients had no awareness of which tests had been done or why, which is at odds with professional guidelines for informed consent.^12^ The lack of information sharing leaves patients reliant on doctors' judgement, advice and expertize, in keeping with a more paternalistic style of medical practice.^13^ These findings are in keeping with video‐recorded UK general practice consultations, where the majority of testing decisions were instigated by doctors, with a lack of information sharing and shared decision‐making.^14^, ^15^

The mismatches in expectation had consequences for patient understanding of test results. ‘Normal’ test results were perceived by doctors to offer reassurance; however, for patients who expected tests to provide answers, normal results could lead to uncertainty, anxiety and a feeling of being dismissed or invalidated. This is in keeping with previous research showing a lack of reassurance from normal test results.^16^ Donovan et al.^17^ found that similar mismatches in doctors' and patients' perspectives were a barrier to patient reassurance in rheumatology clinic settings, with successful reassurance hinging on patient perceptions that their symptoms and problems had been acknowledged.

Shared decision‐making conceptualizes the practice of medicine as a series of discrete choices; this does not seem to fit with the experiences and perspectives of the patients and the clinicians interviewed, who describe tests as one part of a complex medical and social interaction. A broader model of shared understanding seems to be more relevant to the complexity of primary care diagnosis, described by Lehman^18^ as follows:

Clinical care does not consist of a series of easily defined take‐it‐or‐leave it choices but is a process of understanding developed and deepened over time. Sharing understanding with patients is a form of dialogue and interaction which cumulatively develops and which effects changes in both parties: it lies at the heart of primary care, and it is essential for kind and effective clinical practice in all specialties.

The main strength of this study was the ability to compare doctors' and patients' perspectives on the same healthcare encounter, which highlighted mismatches in communication and understanding. The longitudinal nature of the study also allowed us to explore the process of information sharing, in comparison to previous research that has either focussed on decision‐making,^19^ or test result communication.^20^, ^21^

The main limitation is that interviews were based on patients' and GPs' recollection of the healthcare encounter, rather than direct observation of the doctor–patient interaction. This could lead to recall bias; for example, GPs could reinterpret their reasons for testing to rationalize their decision‐making, or overestimate the information that they shared with patients, particularly when interviewed by a fellow GP. Most GPs, however, seemed to be comfortable discussing cases with a fellow clinician with shared understanding and were open about sharing uncertainties rather than appearing defensive. Patients did not appear to be influenced by the researcher's status as a GP and did not query clinical issues or seek alternative clinical views, indicating that they recognized the researcher's role as study interviewer rather than clinician. The benefit of interviewing patients rather than observing consultations is that it allowed us to identify what patients understand and retain after a consultation.

All interviews were conducted in the United Kingdom in the Bristol, North Somerset and South Gloucestershire region, and were limited to those able to speak English; also, the findings may not reflect the processes and expectations of testing in other healthcare systems, or other cultures.

## 5 CONCLUSIONS

Research into shared decision‐making has mostly focussed on treatment decisions; yet, guidelines recommend that these principles of shared decision‐making should be applied to testing decisions.^8^ However, patients cannot develop informed preferences and do not perceive testing decisions as an area where choice is possible, because they do not have access to information and a shared understanding of tests. Without a shared understanding of tests, patients experience frustration, anxiety and uncertainty when test results do not fulfil their expectations.

Our results suggest that improvements to shared understanding of testing are needed, not only at the time of decision‐making but also before, during and after testing. Doctors were concerned that sharing too much information could generate anxiety; however, this was not reflected in patient interviews. Developing a shared understanding of tests was just as important for patients with normal blood test results as abnormal tests. Clinicians who proactively shared their expectations of tests before the results were available were able to improve shared understanding of tests.

Although shared decision‐making is recommended as best practice,^8^ it does not reflect the reality of doctors' and patients' accounts of testing, and can be challenging to implement within the confines of a 10 min consultation.^22^ Communication around testing should promote shared understanding from the initial decision to test through to test results and diagnostic decision‐making. This would align expectations about the meaning and usefulness of tests and lay the foundations for shared decision‐making.

## AUTHOR CONTRIBUTIONS

Jessica Watson, Jonathan Banks, Chris Salisbury, William T. Hamilton and Penny F. Whiting contributed to the conceptualization and design of this project. Interviews and analysis were led by Jessica Watson under the supervision of Jonathan Banks. Emerging results were discussed with all the authors during regular supervision meetings. Jessica Watson wrote the first draft of the paper; all authors contributed to reviewing and editing subsequent drafts and reviewed the final manuscript.

## CONFLICT OF INTEREST

The authors declare no conflict of interest.

## ETHICS STATEMENT

Ethics approval was obtained from the proportionate review subcommittee of the London—Hamstead NHS Research Ethics committee (REC 19/LO/0405).

## Supporting information

Supplementary information.

## Data Availability

The data are not publicly available due to privacy and ethical restrictions. Institutional Review Board conditions of approval for this study state that the data must not be shared with persons not listed on the approved application.
