# Supplementary material for: Blood tests in primary care: A qualitative study of communication and decision‐making between doctors and patients
Source: Health Expect. 2022 Jul 19;25(5):2453–61. doi: 10.1111/hex.13564 (PMC9615068; doi:10.1111/hex.13564)
Supplement: Supplementary file 1 — Supplementary information. [file HEX-25--s001.docx]

# Supplementary material: Topic guide

| **Initial interview with patient participants** | |
| --- | --- |
| **Suggested flow / questions** | **Suggested prompts, if needed** |
| 1. Background. Can you tell me a bit about yourself? | - Work life: What do you do for a living? - Home life: Who is at home with you? |
| 2. Blood test. What prompted you to book a blood test today? | - Did a GP or nurse ask you to book a blood test? - Did you get a call or letter from the practice to book bloods? - Do you know why? |
| 2a. What prompted you to see your GP?  (If blood tests requested following a GP appointment) | - What were you expecting or hoping for from the GP appointment? - Were you hoping or expecting to have blood tests? |
| 2b. Do you know which chronic conditions are being monitored?  (If blood tests being done for routine monitoring) | - Do you know why? |
| 3: Blood tests. What do you know about the blood tests that your GP/nurse requested? | - Do you know which blood tests you are having/have had? - Do you know what these blood tests can pick up? - If inflammatory markers mentioned, then probe further *‘what does ‘inflammatory marker’ mean?’* |
| 4: Communication. What did your GP tell you about your blood tests? | - Did they tell you why they wanted to do blood tests? - Did they give you any options or choices about testing? - Was the decision to test mostly your decision, mostly your GPs decision, or a shared decision? - Did they discuss any benefits or limitations of blood tests? - Did they tell you what the test results might show? - Did they explain how to get the results? - Was there anything else about your blood tests that you wanted to know? - Any sources of information you use to get information about tests? |
| 5: Expectations. What do you expect will happen next? | - Do you know how to get your test results? - Do you know when to get your test results? - How would you like to get your results? - What do you think your test results will tell you? - Do you think the test results will change anything for you? |
| 6: Phlebotomy appointment (if relevant). What was your experience of having your blood test done? | - How easy or difficult was it for you to book an appointment? - How easy or difficult was it for you to have your blood test taken? - Did you discuss your blood tests with the phlebotomist/nurse who took the blood? |
| 7: Suggestions for improvement. Is there anything about your experience of blood testing which could be improved? | - Any suggestions to help doctors and nurses communicate? - Any suggestions to help improve the systems? - Any suggestions for patients? |
| 8: Is there anything else about your blood tests you would like to say that we haven’t mentioned yet? |  |
| **Second interview with patients after blood test results available** | |
| 1. Have you received your test results? | If no test results received:   - How do you feel about that? - Do you know what happens next? - Would you be happy to reschedule a follow up interview? |
| 2. What was your experience of getting your test results? | - How did you get your test results? (telephone, internet, face-to-face?) - Did the practice contact you, or did you call them? - Who gave you the test results? (GP, nurse, receptionist?) - Do you know which blood tests you have had? - Were you given the test results in numbers or just told that they were raised or normal? - What explanation of the meaning of the test results did you receive? - *If test results were abnormal* – what further instructions were you given? (e.g., book appointment with GP, book repeat blood test) - Did you use any other sources of information to find out about your tests? (ego websites, discussions with friends or family) |
| 3. What did your blood test results mean for you? | - Did the results change anything for you? - Do you know what the results mean for your health? - Do you know what happens next? |
| 4. Do you have any suggestions for how communication about testing could be improved? | - What do you want to know about tests? - How do you want to receive information about tests? - Are there any resources you use to find out about tests? - Are there any resources you would like to have about tests? |
| 5. Is there anything else about your blood tests you would like to say that we haven’t mentioned yet? |  |
| **GP interview** | |
| 1. Background. Can you tell me a bit about yourself? | - Years’ experience - Type of practice - Role in the practice (partner/salaried/locum) |
| 1. Patient background. Can you tell me what you remember about your consultation with patient X? | - Why did they come to the doctors? - What do you think they were expecting? - What do you think they were worried about? |
| 1. Choice of test. What prompted you to check bloods on this patient? | - Why did you choose to check inflammatory markers? - What were you looking for? - How do you decide which bloods to check? |
| 1. What were your expectations of the tests? | - What did you think the results would show? |
| 1. Test results. What did the test results show you? | - Did they change your management? - What will you do next? |
| 1. Patient perspectives. What do you think the patient understands about testing? | - Do you think that patient wanted tests done? - Was the decision to do tests mostly your decision, mostly the patients’ decision, or a shared decision? - Do you think the patient knows which tests were done? - What do you think the patient understands about the tests? - What do you think the patient expected from the tests? |
| 1. Communication around testing. What did you explain to the patient? | - What did you explain to the patient about the blood tests you were doing and why? - How will/did you explain the test result? - How do you decide how much information to share with patients? - Are there any resources you use to explain tests to patients? - Is there anything which you would find useful to improve communication around blood testing? |
| 1. Systems of testing. How do systems of testing help or hinder communication with patients? | - Any issues around how the test results are communicated to patients? (text/email/phone calls) - Any issues with communication between hospital/laboratory and primary care of test results? - Any suggestions for improvement to the systems of testing? |
| 1. Is there anything you would like to say that we haven’t mentioned yet? |  |
